# Care Team Members’ Perceptions of an Informatics Intervention to Improve Geriatric Care Across Multiple sites

**DOI:** 10.1093/geroni/igaa057.1675

**Published:** 2020-12-16

**Authors:** Emily Franzosa, Morgan Traylor, Vivian Guerrero Aquino, Kimberly Judon, Ashley Schwartzkopf, Brian Dixon, Kenneth Boockvar

**Affiliations:** 1 James J. Peters VA Medical Center, Brooklyn, New York, United States; 2 Richard L. Roudebush VA Medical Center, Indianapolis, Indiana, United States; 3 VHA, New York, New York, United States; 4 US Department of Veterans Affairs - James J. Peters VA Medical Center, Bronx, New York, United States; 5 Center for Health Information and Communication, Health Services Research & Development Service, Indianapolis, Indiana, United States; 6 James J. Peters VA Medical Center, Bronx, New York, United States

## Abstract

Electronic health information exchange (HIE) may improve care for geriatric patients receiving care across multiple sites by reducing test duplication, medication prescribing errors, and adverse events. This project evaluated the implementation of an HIE intervention at two VA medical centers offering VA providers real-time notification of non-VA inpatient or ED use, followed by post-hospital geriatric care coordination. We interviewed 23 providers (physicians, nurses, social workers and other care team staff) about their experiences with the program. Interviews were analyzed using the Consolidated Framework for Implementation Research (CFIR) to examine 1) goals and expectations for notifications and transitional care; 2) barriers to effective use of notifications and coordination; and 3) suggestions for improvement. Overall, care team members were positive about the intervention, noting it cut down on time searching for outside medical records and that care coordination visits were helpful in answering patients’ questions and clarifying discharge instructions. However, some providers were not aware of the alerts, found the HIE interface challenging to use, or were concerned that expanding the program would create workflow issues. Suggestions for improvements included sharing information about newly prescribed medications, lab and radiological tests, and progress alerts during the episode of care; and including non-VA providers to facilitate care coordination. Social workers also asked to be included on alerts to improve follow-up. Our findings suggest HIE can be a useful tool for coordinating care across sites, provided information can be easily shared between all care team members and HIE interfaces are streamlined to reduce additional work.

